# Proportion of Adverse Events of Injectable Collagen Biostimulators After Facial Aesthetic Treatment: A Systematic Review Protocol

**DOI:** 10.3390/jcm15093182

**Published:** 2026-04-22

**Authors:** Lia Rosana Honnef, Manuella Salm Coelho, Júlia Meller Dias de Oliveira, Helena Polmann, Thaís Marques Simek Vega Gonçalves, Patrícia Pauletto, Cristine Miron Stefani, Victor Ricardo Manuel Munoz-Lora, Graziela De Luca Canto

**Affiliations:** 1 Brazilian Centre for Evidence-Based Research (COBE), Department of Dentistry, Federal University of Santa Catarina (UFSC), Florianópolis 88040-370, Brazil; lia.honnef@hotmail.com (L.R.H.); manuella.salm@gmail.com (M.S.C.); julia_meller5@hotmail.com (J.M.D.d.O.); hpolmann@hotmail.com (H.P.); thaisgonc@gmail.com (T.M.S.V.G.); cmstefani@gmail.com (C.M.S.); delucacanto@gmail.com (G.D.L.C.); 2 School of Dentistry, Universidad De Las Américas (UDLA), Quito E9-241, Ecuador; 3 Department of Dentistry, Health Sciences School, University of Brasilia (UNB), Brasília 70910-900, Brazil; 4 Department of Facial Aesthetics, Guarulhos University, São Paulo 07023-070, Brazil; victormunoz2512@gmail.com; 5 Postgraduate Program in Evidence-Based Health, Department of Medicine, Federal University of São Paulo, São Paulo 04024-002, Brazil

**Keywords:** systematic review, collagen biostimulator, aesthetic treatment, complications, adverse effects

## Abstract

Background: With the increasing demand for non-surgical facial rejuvenation, injectable collagen biostimulators such as poly-L-lactic acid (PLLA), calcium hydroxyapatite (CaHA), polycaprolactone (PCL), poly-D,L-lactic acid (PDLLA) and powdered polydioxanone (PPDO) have become widely used by facial aesthetic practitioners. These agents stimulate neocollagenesis, providing gradual improvement in skin firmness, elasticity and facial contour with long-lasting results. While manufacturers emphasize the efficacy and favorable safety profile of these products, adverse events such as nodules, edema, inflammatory reactions and, in rare cases, granulomas have been reported. To date, no comprehensive systematic review has evaluated the proportion and nature of adverse effects associated with all major collagen biostimulators in facial aesthetic procedures. This study aims to synthesize current evidence on the proportion of adverse events linked to injectable collagen biostimulators. Methods: The systematic review will include clinical studies involving adults undergoing facial aesthetic procedures with PLLA, PDLLA, CaHA, PCL and PPDO that report adverse events during or after treatment. The search will be conducted in six main databases: CENTRAL, EMBASE, LILACS, PubMed, SCOPUS and Web of Science. No restrictions will be applied regarding language or publication date. The screening process will occur in two phases: first, two independent reviewers will assess titles and abstracts against the eligibility criteria; second, the same reviewers will conduct full-text evaluations. Data will be synthesized narratively, with a meta-analysis of proportions performed if appropriate. Additionally, sample characteristics, treatment protocols, study design and main findings will be reported. The risk of bias will be assessed independently by two reviewers using appropriate tools, based on the study design, with the support of artificial intelligence. PROSPERO registration number: CRD420251062785.

## 1. Background

Facial aging is a complex, multifactorial process characterized by progressive changes in the skin, muscular, adipose and skeletal components of the face, leading to volume loss, skin laxity and diminished elasticity [1,2,3]. In response to the growing demand for minimally invasive rejuvenation procedures, injectable collagen biostimulators, particularly poly-L-lactic acid (PLLA), calcium hydroxyapatite (CaHA), polycaprolactone (PCL), poly-D,L-lactic acid (PDLLA) and powdered polydioxanone (PPDO) have been increasingly incorporated into clinical practice [4]. These agents induce neocollagenesis through a controlled inflammatory response, primarily stimulating the production of type I collagen, which contributes to dermal thickening, provides structural support and improves skin quality over time [5].

The increasing popularity of non-surgical aesthetic procedures has led to a substantial rise in the use of biostimulators by facial aesthetic practitioners. While these agents demonstrate consistent efficacy and a generally favorable safety profile, adverse events such as erythema, edema, injection-site pain, nodule formation, hypersensitivity and, in rare cases, granulomatous reactions have been reported [6]. These complications, although often mild and self-limiting, can compromise aesthetic outcomes, affect patient satisfaction and occasionally require medical intervention.

While several systematic reviews have addressed specific aspects of injectable fillers, they present significant limitations in scope and methodology. One study [7] focused exclusively on PLLA, including only randomized controlled trials related to facial aging and lipoatrophy, thereby excluding real-world data and adverse events from broader populations. Similarly, another one evaluated non-hyaluronic acid fillers but restricted their search to PubMed and Embase, with a narrow focus on midface augmentation [8]. Another review concentrated exclusively on PLLA, without evaluating CaHA and PCL, and combined heterogeneous clinical endpoints without a focused assessment of adverse events [9]. Furthermore, one network meta-analysis centered on nasolabial fold treatment outcomes rather than a comprehensive evaluation of biostimulator safety profiles [10]. Lastly, one review focused solely on CaHA and PCL, excluding PLLA entirely from their safety assessment [6].

In contrast, our systematic review protocol aims to provide the most comprehensive synthesis to date of adverse events associated with five commonly used injectable collagen biostimulators for facial aesthetics: PLLA, PDLLA, CaHA, PCL and PPDO. This comprehensive approach is designed to inform clinical decision-making, enhance patient safety and improve the quality of patient counseling regarding the risks associated with the use of biostimulators in facial rejuvenation. The objective of this systematic review is to answer the following specific question: “What is the proportion of patients experiencing adverse events after facial aesthetic treatment with injectable collagen biostimulators?”.

## 2. Methods

### 2.1. Protocol and Registration

This protocol was developed in accordance with the Preferred Reporting Items for Systematic Reviews and Meta-Analyses Protocols (PRISMA-P) [11] (File S1). The protocol has been registered in the International Prospective Register of Systematic Reviews (PROSPERO) under the number: CRD420251062785 [12,13,14].

### 2.2. Research Question

The research question is based on the PIOS acronym (Table 1) [12,13,15,16].

### 2.3. Eligibility Criteria

#### 2.3.1. Inclusion Criteria

The following inclusion criteria will be applied:

- Studies with adults (≥18 years old);
- Studies that assessed injected collagen biostimulators for facial aesthetic purposes, such as PLLA, PDLLA, CaHA, PCL, PPDO and hybrid filler (CaHA with hyaluronic acid), and reported possible adverse effects during and/or after treatment;
- Randomized clinical trials, non-randomized clinical trials, quasi-randomized clinical trials (before and after) and prospective observational studies.

Note: In the case of randomized and non-randomized clinical studies, information will be collected from the group of patients who received collagen biostimulator injections for facial aesthetic purposes (single-arm).

#### 2.3.2. Exclusion Criteria

The following studies will be excluded:

(1) Studies in which the sample was exclusively composed of specific groups, such as participants with systemic diseases, syndromes, or autoimmune diseases;
(2) Studies that used collagen biostimulators for purposes other than facial aesthetics (e.g., only body treatment), or that used them with other therapies (co-interventions) and isolating results was not possible, or studies that used substances other than collagen biostimulators (e.g., polymethyl methacrylate);
(3) Pre-clinical studies;
(4) Descriptive studies, reviews, letters, books, conference abstracts, case reports, opinion articles, technique articles, posters, retrospective observational studies and clinical practice guidelines;
(5) Full text is unavailable, even after contacting the corresponding authors (three attempts in 3 weeks).

### 2.4. Information Sources and Search Strategy

A comprehensive search strategy was developed and tested by the authors, with the support of a specialized librarian in health sciences (File S2) on PubMed on 28 July 2025. An update will be performed in April 2026. The systematic search will be conducted in the six following databases from inception to the present: Cochrane Central Register of Controlled Trials in the Cochrane Library (CENTRAL), EMBASE (Elsevier) (1974 to present), LILACS via BVS (in Spanish: *Literatura Latinoamericana y del Caribe en Ciencias de la Salud*, 1982 to present), MEDLINE via PubMed (1946 to present), SCOPUS (Elsevier) and Web of Science (Clarivate Analytics/Thomson Reuters). The grey literature will be consulted through Google Scholar, the first 200 references will be retrieved, as recommended by Haddaway et al. (2015) [17], and ProQuest Dissertations & Theses Index. The citation list of the included studies and experts in the subject will be consulted. Artificial intelligence (AI) research tools, such SciSpace (SciSpace, OpenAI, 2022), will be utilized to identify additional studies [12,18].

### 2.5. Study Records

#### 2.5.1. Data Management

The records retrieved from each database will be imported into a reference management software (EndNote Web™, Clarivate™, Philadelphia, PA, USA), where duplicates will be identified and removed. Subsequently, the records will be uploaded to an artificial intelligence–assisted platform for systematic reviews to support phase 1 title and abstract screening (Laser AI, Evidence Prime, Hamilton, ON, Canada) [13,16,18,19,20]. Phase 2 full-text screening will be conducted using a dedicated screening platform (Rayyan^®^, Qatar Computing Research Institute, Doha, Qatar). All screening decisions will be performed by two independent reviewers.

#### 2.5.2. Methods for Study Selection

Study selection will be conducted in two phases by two calibrated and independent reviewers (L.R.H. and M.S.C.) guided by predefined inclusion and exclusion criteria. In phase 1, the reviewers will independently screen titles and abstracts of the retrieved records. In phase 2, the same reviewers will independently assess the full texts of studies deemed potentially eligible. The same eligibility criteria will be applied in both phases. Any disagreements will be resolved through discussion and if necessary, by consultation with a third reviewer (J.M.D.d.O.) [12,14,18,19].

#### 2.5.3. Data Collection Process

Data extraction will be conducted using a standardized, pre-designed form. Artificial intelligence tools (ChatGPT, GPT-4 version; OpenAI, 2024) will be used solely to assist in the identification and organization of relevant information from the included studies, without replacing human judgment. The prompts used for this purpose will be provided in the Supplementary Material to ensure transparency and reproducibility. All extracted data will be independently verified by two authors (L.R.H. and M.S.C.) and discrepancies will be resolved by consensus. The finalized data will be entered into a pre-designed Microsoft^®^ Excel form (version 16.29.1; Microsoft Office 2019, Microsoft, Redmond, WA, USA). The extracted data will be presented in tabular format summarizing the characteristics of the included studies. When two or more records originate from the same study, they will be grouped and analyzed as a single dataset [12,14,18,19].

#### 2.5.4. Data Items

From the studies found, the following data will be collected: Authors, country, publication year, sample size, sex, mean age and standard deviations or age range, collagen biostimulator formulation used, its dilution, treated facial region, sessions quantity, dose, technique, follow-up period, collagen biostimulator adverse event type and its frequency, detection method criteria for adverse effects and industry funding [12,14,18,19]. If a primary study does not directly report the proportion of patients experiencing adverse events, it will be calculated by extracting the number of cases and the total sample size from the available data. The number of individuals exhibiting ≥1 event of interest will be used as the numerator and the total number of participants assessed in the study will serve as the denominator.

#### 2.5.5. Outcomes and Prioritization

The main outcome will be any adverse event associated with the intervention. The outcome must have been determined through clinical evaluation conducted by healthcare professionals and/or through patient-reported outcomes, collected either via standardized questionnaires or through spontaneous participant reporting. Adverse events will be classified based on their time of onset [21]: (1) Immediate events, occurring within the first 24 h post-procedure, including manifestations such as erythema, edema, pain, bruising, pruritus, vascular occlusion, tissue necrosis and visual impairment; (2) Early events, developing between 24 h and 4 weeks after the procedure, encompassing complications such as infection, abscess formation, herpes simplex virus outbreaks, hypersensitivity reactions and the appearance of non-inflammatory nodules or palpable lumps; and (3) Delayed events, arising more than 4 weeks post-intervention, including persistent or malar edema, delayed hypersensitivity reactions, Tyndall effect, foreign-body granuloma formation, biofilm-associated infections and late-onset inflammatory nodules. This standardized temporal categorization aims to ensure consistency in the reporting and interpretation of adverse events across studies [12,14,18,19]. Whenever the primary studies provide sufficient information, adverse events will also be categorized according to their clinical severity (e.g., mild, moderate, or severe).

### 2.6. Risk of Bias in Individual Studies

Two independent reviewers (L.R.H. and M.S.C.) will assess the risk of bias in the included studies. The tools used will be chosen based on the type of study included: RoB 2.0 [22] for randomized clinical trials and ROBINS-I [23] for non-randomized studies of intervention (more information available at www.riskofbias.info (accessed on 6 April 2025)). Differences in the evaluation will be resolved by a third reviewer (J.M.D.d.O.).

Before applying the tool, the authors will discuss it and establish the evaluation parameters. Subsequently, a calibration session between the evaluators will be conducted. AI, specifically (ChatGPT, GPT-4 version; OpenAI, 2024), will assist in this process by answering domain-specific questions for each tool based on predefined commands. These prompts will be made available in the Supplementary Material to enhance transparency and reproducibility. Importantly, ChatGPT will not make final risk-of-bias judgments. As a verification step, the two authors (L.R.H. and M.S.C.) will independently review the AI-extracted data. The results will be presented as recommended by each tool. Figures will be created on the robvis tool website (https://www.riskofbias.info/welcome/robvis-visualization-tool (accessed on 6 April 2026)) [12,14,18,19].

### 2.7. Data Synthesis

The research team plans to use the PERSyst MA version 1.0 (https://persyst.group/persystma/ (accessed on 6 April 2026)), an online application for R package meta, version 7.0-0, to conduct a meta-analysis of proportions. Pooled proportions will represent the cumulative incidence (risk) of patients experiencing the event during study follow-up (e.g., patients with ≥1 event/total treated). Subgroup analysis will be conducted by follow-up window and/or by timing of event onset (immediate, early, delayed), when feasible. Other planned subgroup analyses encompass adverse event type and collagen biostimulator formulation. Adverse events will be synthesized as: (i) any adverse event (≥1 event per patient) and (ii) specific adverse event types, when data allow. Meta-analyses will be performed separately by event type and may be grouped according to onset timing. Clinical and methodological considerations will be applied to determine the pertinence of meta-analyses [18]. Pooled estimates and 95% confidence intervals will be calculated using a random-effects model and a random-intercepts logistic regression, with the logit transformation applied to the proportions (Generalized Linear Mixed Model—GLMM). This method is recommended for meta-analysing the proportion of rare events and reduces the risk of misleading results from back-transforming more common stabilizing methods, such as the Freeman-Tuckey Double Arcsine method [24]. Heterogeneity will be quantified using maximum likelihood as the variance estimator [25].

Studies reporting zero events will not be excluded. When feasible, zero-event studies will be incorporated directly into the meta-analysis model, since generalized linear mixed models can accommodate such data without requiring continuity corrections in many cases. If sparse-data issues prevent model convergence, this will be reported and addressed through appropriate sensitivity analyses.

### 2.8. Meta-Bias(es)

An exhaustive literature search will be conducted to minimize the risk of publication bias. In addition, to uphold the research’s integrity and impartiality, affiliations of the sponsors associated with the included studies will be assessed and any potential conflicts of interest among the authors will be scrutinized. Publication bias will be assessed through funnel plots and Egger’s test if ten or more articles are included [26].

### 2.9. Confidence in Cumulative Evidence

The certainty of evidence will not be assessed. Given that this review focuses exclusively on adverse events and includes meta-analyses of proportions, the GRADE approach may not be directly applicable, as several of its domains were primarily developed for comparative intervention outcomes and require methodological adaptations when applied to harm outcomes without effect estimates or comparator groups. Therefore, in the absence of standardized guidance for this context, certainty of evidence assessment will not be performed [27,28].

### 2.10. Heterogeneity

Statistical heterogeneity among the results of different trials will be assessed using the Chi-square (Chi^2^) test, with significance defined as *p* < 0.1. The I^2^ statistic will be used to assess heterogeneity unexplained by chance alone across studies in each analysis. Values above 50% will be considered relevant and reasons for heterogeneity will be explored through subgroup analysis. Consistency will be explored by the calculation of prediction intervals. The impact of high-risk-of-bias studies will be investigated through sensitivity analysis, by removing studies with high risk of bias from each meta-analysis [12,14,18].

## 3. Discussion

This systematic review aims to rigorously synthesize the current evidence on adverse events associated with injectable collagen biostimulators in facial aesthetic procedures. The study will be characterized by a robust and transparent methodology, including comprehensive searches across six major databases, grey literature, manual searches of reference lists and consultation with field experts. This comprehensive strategy is designed to minimize publication bias and maximize the accuracy and generalizability of the results.

The methodological rigor, including independent screening by two blinded reviewers and arbitration by a third reviewer, is expected to ensure reproducibility and reduce selection bias. This approach will support the development of reliable synthesis of available data on the proportion, severity and types of adverse events related to PLLA, PDLLA, CaHA, PCL and PPDO.

The findings from this review will be particularly valuable for informing clinical practice, improving the accuracy of risk communication with patients and potentially guiding future regulatory and educational policies regarding injectable collagen biostimulators. Despite anticipated limitations, such as the heterogeneity of study designs and varying quality of available evidence, this systematic review is expected to make a significant contribution to the understanding of the safety profile of these agents, thereby fostering a balanced and evidence-based approach in facial aesthetic medicine.

## Figures and Tables

**Table 1:** Research question based on the PIOs acronym.

P	PARTICIPANTS	Adults (≥18 Years Old).
I	INTERVENTION	Facial aesthetic treatment with injectable collagen biostimulators.
O	OUTCOMES	Proportion of adverse events during follow-up.
S	STUDY DESIGN	Clinical trials, randomized or not, before-and-after studies and observational studies.

## Data Availability

The data are fully available in the paper.

## Supplementary Materials

The following supporting information can be downloaded at: https://www.mdpi.com/article/10.3390/jcm15093182/s1, File S1: PRISMA-P (Preferred Reporting Items for Systematic review and Meta-Analysis Protocols) 2015 checklist: recommended items to address systematic review protocol. File S2: Search Strategy.

## Abbreviations

CaHA	Calcium Hydroxyapatite.
CENTRAL	Cochrane Central Register of Controlled Trials in the Cochrane Library.
PCL	Polycaprolactone.
PDLLA	Poly-D:L-Lactic Acid.
PLLA	Poly-L-Lactic Acid.
PPDO	Powdered Polydioxanone.
PRISMA-P	Preferred Reporting Items for Systematic Reviews and Meta-Analyses Protocols.
PROSPERO	International prospective register of systematic reviews.
LILACS	Literatura Latinoamericana y del Caribe en Ciencias de la Salud.
