# Supplementary material for: Proportion of Adverse Events of Injectable Collagen Biostimulators After Facial Aesthetic Treatment: A Systematic Review Protocol
Source: J Clin Med. 2026 Apr 22;15(9):3182. doi: 10.3390/jcm15093182 (PMC13163661; doi:10.3390/jcm15093182)
Supplement: Supplementary file 1 [file jcm-15-03182-s001.zip › File S2.pdf]

FILE S2 - Search Strategy

DATABASE	QUERY SEARCH	RESULTS
PUBMED	#1 AND #2 AND #3	625
3	("adverse effects"[Title/Abstract] OR "adverse events"[Title/Abstract] OR "Adverse reaction"[Title/Abstract] OR "complication"[Title/Abstract] OR "complications"[Title/Abstract] OR "nodule"[Title/Abstract] OR "nodules"[Title/Abstract] OR "papule"[Title/Abstract] OR "dyschromia"[Title/Abstract] OR "vascular compression"[Title/Abstract] OR "Edema"[MeSH Terms] OR "Edema"[Title/Abstract] OR "Dropsy"[Title/Abstract] OR "Hydrops"[Title/Abstract] OR "Anasarca"[Title/Abstract] OR "Contusions"[MeSH Terms] OR "contusion*"[Title/Abstract] OR "bruis*"[Title/Abstract] OR "Hematoma"[MeSH Terms] OR "hematoma*"[Title/Abstract] OR "Erythema"[MeSH Terms] OR "erythema*"[Title/Abstract] OR "Ecchymosis"[MeSH Terms] OR "ecchymos*"[Title/Abstract] OR "Pruritus"[MeSH Terms] OR "Pruritus"[Title/Abstract] OR "itching"[Title/Abstract] OR "Pruritis"[Title/Abstract] OR "Pain"[MeSH Terms] OR "Pain"[Title/Abstract] OR "Ache"[Title/Abstract] OR "Aches"[Title/Abstract] OR "physical suffering*"[Title/Abstract] OR "Ulcer"[MeSH Terms] OR "ulcer*"[Title/Abstract] OR "Infections"[MeSH Terms] OR "infection*"[Title/Abstract] OR "Exanthema"[MeSH Terms] OR "exanthem*"[Title/Abstract] OR "rash"[Title/Abstract] OR "Keloid"[MeSH Terms] OR "Keloid"[Title/Abstract] OR "Granuloma"[MeSH Terms] OR "Granuloma"[Title/Abstract] OR "Cellulitis"[MeSH Terms] OR "Cellulitis"[Title/Abstract] OR "Necrosis"[MeSH Terms] OR "Necrosis"[Title/Abstract] OR "Blindness"[MeSH Terms] OR "vision loss"[Title/Abstract] OR "Blindness"[Title/Abstract] OR "Anaphylaxis"[MeSH Terms] OR "Anaphylaxis"[Title/Abstract] OR "Hypersensitivity"[MeSH Terms] OR "Hypersensitivity"[Title/Abstract] OR "Hypersensitivities"[Title/Abstract] OR "Allergy"[Title/Abstract] OR "Allergies"[Title/Abstract] OR "Allergic Reaction"[Title/Abstract] OR "Allergic Reactions"[Title/Abstract])	7,763,537
2	"Polydioxanone"[Mesh] OR "polydioxanone"[Title/Abstract] OR "PDO filler"[Title/Abstract] OR "Ultra V"[Title/Abstract] OR "ULTRACOL"[Title/Abstract] OR "Poly-D,L-lactic acid"[Title/Abstract] OR "PDLLA"[Title/Abstract] OR "AestheFill"[Title/Abstract] OR "Durapatite"[MeSH Terms] OR "Durapatite"[Title/Abstract] OR "Hydroxyapatite"[Title/Abstract] OR "Hydroxylapatite"[Title/Abstract] OR "Interpore 200"[Title/Abstract] OR "Interpore200"[Title/Abstract] OR "Interpore 500"[Title/Abstract] OR "Interpore500"[Title/Abstract] OR "Alveograf"[Title/Abstract] OR "Calcitite"[Title/Abstract] OR "Osprovit"[Title/Abstract] OR "Ossopan"[Title/Abstract] OR "Osteogen"[Title/Abstract] OR "Periograf"[Title/Abstract] OR "polycaprolactone"[Supplementary Concept] OR "polycaprolactone"[Title/Abstract] OR "poly lactide"[Supplementary Concept] OR "poly-L-lactic acid"[Title/Abstract] OR "PLLA"[Title/Abstract] OR "polylactic acid"[Title/Abstract] OR "Radiesse"[Title/Abstract] OR "rennova"[Title/Abstract] OR "ellanse"[Title/Abstract] OR "harmonyca"[Title/Abstract] OR "Sculptra"[Title/Abstract] OR "Elleva"[Title/Abstract] OR "Diamond"[Title/Abstract] OR "collagen biostimulat*"[Title/Abstract] OR	87,681

	"CaHA"[Title/Abstract] OR (("Collagen"[MeSH Terms] OR "Collagen"[Title/Abstract]) AND "biostimulat*"[Title/Abstract])	
1	("Dermal Fillers"[MeSH Terms] OR "dermal filler*"[Title/Abstract] OR "skin filler*"[Title/Abstract] OR "injections, subcutaneous"[MeSH Terms] OR "subcutaneous injection*"[Title/Abstract] OR "Cosmetic Techniques"[MeSH Terms] OR "cosmetic technique*"[All Fields] OR "cosmetic technic*"[All Fields])	125,101
EMBASE	('adverse effects':ab,ti,kw OR 'adverse events':ab,ti,kw OR 'adverse reaction':ab,ti,kw OR complication:ab,ti,kw OR complications:ab,ti,kw OR nodule:ab,ti,kw OR nodules:ab,ti,kw OR papule:ab,ti,kw OR dyschromia:ab,ti,kw OR 'vascular compression':ab,ti,kw OR 'edema'/exp OR edema:ab,ti,kw OR dropsy:ab,ti,kw OR hydrops:ab,ti,kw OR anasarca:ab,ti,kw OR 'contusion'/exp OR contusion*:ab,ti,kw OR bruis*:ab,ti,kw OR 'hematoma'/exp OR hematoma*:ab,ti,kw OR 'erythema'/exp OR erythema*:ab,ti,kw OR 'ecchymosis'/exp OR ecchymos*:ab,ti,kw OR 'pruritus'/exp OR pruritus:ab,ti,kw OR itching:ab,ti,kw OR pruritis:ab,ti,kw OR 'pain'/exp OR pain:ab,ti,kw OR ache:ab,ti,kw OR aches:ab,ti,kw OR 'physical suffering*':ab,ti,kw OR 'ulcer'/exp OR ulcer*:ab,ti,kw OR 'infection'/exp OR infection*:ab,ti,kw OR 'exanthema'/exp OR exanthem*:ab,ti,kw OR rash:ab,ti,kw OR 'keloid'/exp OR keloid:ab,ti,kw OR 'granuloma'/exp OR granuloma:ab,ti,kw OR 'cellulitis'/exp OR cellulitis:ab,ti,kw OR 'necrosis'/exp OR necrosis:ab,ti,kw OR 'blindness'/exp OR blindness:ab,ti,kw OR 'vision loss':ab,ti,kw OR 'anaphylaxis'/exp OR anaphylaxis:ab,ti,kw OR 'hypersensitivity'/exp OR hypersensitivity:ab,ti,kw OR hypersensitivities:ab,ti,kw OR allergy:ab,ti,kw OR allergies:ab,ti,kw OR 'allergic reaction':ab,ti,kw OR 'allergic reactions':ab,ti,kw) AND ('polydioxanone'/exp OR polydioxanone:ti,ab,kw OR "PDO filler":ti,ab,kw OR "Ultra V":ti,ab,kw OR ULTRACOL:ti,ab,kw OR "poly-D,L-lactic acid":ti,ab,kw OR PDLLA:ti,ab,kw OR AestheFill:ti,ab,kw OR 'durapatite'/exp OR durapatite:ab,ti,kw OR hydroxyapatite:ab,ti,kw OR hydroxylapatite:ab,ti,kw OR 'interpore 200':ab,ti,kw OR interpore200:ab,ti,kw OR 'interpore 500':ab,ti,kw OR interpore500:ab,ti,kw OR alveograf:ab,ti,kw OR calcitite:ab,ti,kw OR osprovit:ab,ti,kw OR ossopan:ab,ti,kw OR osteogen:ab,ti,kw OR periograf:ab,ti,kw OR polycaprolactone:ab,ti,kw OR 'poly lactide':ab,ti,kw OR 'poly-l-lactic acid':ab,ti,kw OR plla:ab,ti,kw OR 'polylactic acid':ab,ti,kw OR radiesse:ab,ti,kw OR rennova:ab,ti,kw OR ellanse:ab,ti,kw OR harmonyca:ab,ti,kw OR sculptra:ab,ti,kw OR elleva:ab,ti,kw OR diamond:ab,ti,kw OR 'collagen biostimulat*':ab,ti,kw OR (collagen:ab,ti,kw AND biostimulat*:ab,ti,kw)) AND ('dermal filler'/exp OR 'dermal filler*':ab,ti,kw OR 'skin filler*':ab,ti,kw OR 'subcutaneous injection'/exp OR 'subcutaneous injection*':ab,ti,kw OR 'cosmetic technique' OR 'cosmetic technique*':ab,ti,kw OR 'cosmetic technic*':ab,ti,kw)	468
LILACS via BVS	(tw:"adverse effects" OR tw:"efeitos adversos" OR tw:"efectos adversos" OR tw:"adverse events" OR tw:"eventos adversos" OR tw:"eventos adversos" OR tw:"adverse reaction" OR tw:"reação adversa" OR	11

	tw:"reacciones adversas" OR tw:complication OR tw:complicações OR tw:complicaciones OR tw:nodule OR tw:nódulo OR tw:nódulo OR tw:nodules OR tw:nódulos OR tw:nódulos OR tw:papule OR tw:pápula OR tw:pápula OR tw:dyschromia OR tw:discromia OR tw:discromía OR tw:"vascular compression" OR tw:"compressão vascular" OR tw:"compresión vascular" OR mh:"Edema" OR tw:edema OR tw:dropsy OR tw:hidropsia OR tw:anasarca OR mh:"Contusions" OR tw:contusion* OR tw:contusão OR tw:contusión OR tw:bruis* OR tw:hematoma* OR mh:"Hematoma" OR tw:hematoma OR mh:"Erythema" OR tw:erythema* OR tw:eritema OR tw:eritema OR mh:"Ecchymosis" OR tw:ecchymos* OR tw:equinose OR tw:equimosis OR mh:"Pruritus" OR tw:pruritus OR tw:prurido OR tw:prurito OR tw:itching OR tw:pruritis OR mh:"Pain" OR tw:pain OR tw:dor OR tw:dolor OR tw:ache OR tw:aches OR tw:"physical suffering*" OR tw:"sofrimento físico" OR tw:"sufrimiento físico" OR mh:"Ulcer" OR tw:ulcer* OR tw:úlcera OR tw:úlcera OR mh:"Infections" OR tw:infection* OR tw:infecção OR tw:infección OR mh:"Exanthema" OR tw:exanthem* OR tw:exantema OR tw:exantema OR tw:rash OR tw:erupção OR tw:erupción OR mh:"Keloid" OR tw:keloid OR tw:queloide OR tw:queloide OR mh:"Granuloma" OR tw:granuloma OR mh:"Cellulitis" OR tw:cellulitis OR tw:celulite OR tw:celulitis OR mh:"Necrosis" OR tw:necrosis OR tw:necrose OR mh:"Blindness" OR tw:blindness OR tw:cegueira OR tw:ciegueira OR tw:"vision loss" OR tw:"perda de visão" OR tw:"pérdida de visión" OR mh:"Anaphylaxis" OR tw:anaphylaxis OR tw:anafilaxia OR mh:"Hypersensitivity" OR tw:hypersensitivity OR tw:hipersensibilidade OR tw:hipersensibilidad OR tw:hypersensitivities OR tw:allergy OR tw:alergia OR tw:allergies OR tw:alergias OR tw:"allergic reaction" OR tw:"reação alérgica" OR tw:"reacción alérgica" OR tw:"allergic reactions" OR tw:"reações alérgicas" OR tw:"reacciones alérgicas") AND (tw:"polydioxanone" OR tw:"polidioxanona" OR tw:"polidioxanona líquida" OR tw:"PDO filler" OR tw:"relleno PDO" OR tw:"preenchimento PDO" OR tw:"Ultra V" OR tw:"ULTRACOL" OR tw:"poly-D,L-lactic acid" OR tw:"ácido poli-D,L-láctico" OR tw:"PDLLA" OR tw:"AestheFill" OR tw:"Durapatite" OR tw:"Hydroxyapatite" OR tw:"Hidroxiapatita" OR tw:"Hidroxiapatita" OR tw:"Hydroxylapatite" OR tw:"polycaprolactone" OR tw:"policaprolactona" OR tw:"poly lactide" OR tw:"ácido poli-L-láctico" OR tw:"ácido poli-L-láctico" OR tw:"poly-L-lactic acid" OR tw:"PLLA" OR tw:"polylactic acid" OR tw:"ácido polilático" OR tw:"Radiesse" OR tw:"collagen biostimulat*" OR tw:"biestimulador de colágeno" OR tw:"bioestimulador de colágeno" OR tw:"bioestimulador de colágeno" OR (tw:"collagen" OR tw:"colágeno" OR tw:"colágeno") AND tw:"biostimulat*" OR tw:"bioestimulador*" OR tw:"biestimulador*") AND (mh:"Dermal Fillers" OR tw:"dermal filler*" OR tw:"preenchimento dérmico" OR tw:"relleno dérmico" OR tw:"skin filler*" OR mh:"Injections, Subcutaneous" OR tw:"subcutaneous injection*" OR tw:"injeções subcutâneas" OR tw:"inyecciones subcutáneas" OR mh:"Cosmetic Techniques" OR tw:"cosmetic technique*" OR tw:"técnicas cosméticas" OR tw:"técnicas cosméticas" OR tw:"cosmetic technic*" OR tw:"técnica cosmética" OR tw:"técnica cosmética") AND db:("LILACS") AND instance:"regional"	
Cochrane	((dermal NEXT filler) OR (skin NEXT filler) OR (subcutaneous NEXT injection) OR (cosmetic NEXT technique) OR (cosmetic NEXT technic))	78

	AND ("polydioxanone" OR (PDO NEXT filler) OR (Ultra NEXT V) OR "ULTRACOL" OR (poly-D,L-lactic NEXT acid) OR "PDLLA" OR "AestheFill" OR "Durapatite" OR "Hydroxyapatite" OR "Hydroxylapatite" OR "Interpore 200" OR "Interpore200" OR "Interpore 500" OR "Interpore500" OR "Alveograf" OR "Calcitite" OR "Osprovit" OR "Ossopan" OR "Osteogen" OR "Periograf" OR "polycaprolactone" OR "poly lactide" OR "poly-L-lactic acid" OR "PLLA" OR "polylactic acid" OR "Radiesse" OR "rennova" OR "ellanse" OR "harmonyca" OR "Sculptra" OR "Elleva" OR "Diamond" OR (collagen NEXT biostimulat) OR "CaHA") AND ("adverse effects" OR "adverse events" OR "adverse reaction" OR "complication" OR "complications" OR "nodule" OR "nodules" OR "papule" OR "dyschromia" OR (vascular NEXT compression) OR "edema" OR "dropsy" OR "hydrops" OR "anasarca" OR "contusion" OR "bruis" OR "hematoma" OR "erythema" OR "ecchymos" OR "pruritus" OR "itching" OR "pruritis" OR "pain" OR "ache" OR "aches" OR (physical NEXT suffering) OR "ulcer" OR "infection" OR "exanthem" OR "rash" OR "keloid" OR "granuloma" OR "cellulitis" OR "necrosis" OR "blindness" OR (vision NEXT loss) OR "anaphylaxis" OR "hypersensitivity" OR "hypersensitivities" OR "allergy" OR "allergies" OR (allergic NEXT reaction) OR (allergic NEXT reactions))	
SCOPUS	TITLE-ABS-KEY("adverse effects" OR "adverse events" OR "adverse reaction" OR complication OR complications OR nodule OR nodules OR papule OR dyschromia OR "vascular compression" OR edema OR dropsy OR hydrops OR anasarca OR contusion* OR bruis* OR hematoma* OR erythema* OR ecchymos* OR pruritus OR itching OR pruritis OR pain OR ache OR aches OR "physical suffering*" OR ulcer* OR infection* OR exanthem* OR rash OR keloid OR granuloma OR cellulitis OR necrosis OR blindness OR "vision loss" OR anaphylaxis OR hypersensitivity OR hypersensitivities OR allergy OR allergies OR "allergic reaction" OR "allergic reactions") AND TITLE-ABS-KEY ("polydioxanone" OR "PDO filler" OR "Ultra V" OR "ULTRACOL" OR "poly-D,L-lactic acid" OR "PDLLA" OR "AestheFill" OR "Durapatite" OR "Hydroxyapatite" OR "Hydroxylapatite" OR "Interpore 200" OR "Interpore200" OR "Interpore 500" OR "Interpore500" OR "Alveograf" OR "Calcitite" OR "Osprovit" OR "Ossopan" OR "Osteogen" OR "Periograf" OR "polycaprolactone" OR "poly lactide" OR "poly-L-lactic acid" OR "PLLA" OR "polylactic acid" OR "Radiesse" OR "rennova" OR "ellanse" OR "harmonyca" OR "Sculptra" OR "Elleva" OR "Diamond" OR "collagen biostimulat*" OR (collagen AND biostimulat*)) AND TITLE-ABS-KEY("dermal filler*" OR "skin filler*" OR "subcutaneous injection*" OR "cosmetic technique*" OR "cosmetic technic*")	789
Web of Science	TS=("adverse effect" OR "adverse effects" OR "adverse event" OR "adverse events" OR "adverse reaction" OR "adverse reactions" OR complication OR complications OR nodule OR nodules OR papule OR papules OR dyschromia OR "vascular compression" OR edema OR dropsy OR hydrops OR anasarca OR contusion OR contusions OR bruise OR bruises OR hematoma OR hematomas OR erythema OR erythemas OR ecchymosis OR ecchymoses OR pruritus OR pruritus OR itching OR itchings OR pain OR pains OR ache OR aches OR "physical suffering" OR ulcer OR ulcers OR infection OR infections OR exanthema OR exanthemas	561

	OR rash OR rashes OR keloid OR keloids OR granuloma OR granulomas OR cellulitis OR cellulitides OR necrosis OR necroses OR blindness OR "vision loss" OR anaphylaxis OR anaphylaxes OR hypersensitivity OR hypersensitivities OR allergy OR allergies OR "allergic reaction" OR "allergic reactions") AND TS=("polydioxanone" OR "PDO filler" OR "Ultra V" OR "ULTRACOL" OR "poly-D,L-lactic acid" OR "PDLLA" OR "AestheFill" OR "Durapatite" OR "Hydroxyapatite" OR "Hydroxylapatite" OR "Interpore 200" OR "Interpore200" OR "Interpore 500" OR "Interpore500" OR "Alveograf" OR "Calcitite" OR "Osprovit" OR "Ossopan" OR "Osteogen" OR "Periograf" OR "polycaprolactone" OR "poly lactide" OR "poly-L-lactic acid" OR "PLLA" OR "polylactic acid" OR "Radiesse" OR "rennova" OR "ellanse" OR "harmonyca" OR "Sculptra" OR "Elleva" OR "Diamond" OR "collagen biostimulator" OR "collagen biostimulators" OR collagen) AND TS=("dermal filler" OR "dermal fillers" OR "skin filler" OR "skin fillers" OR "subcutaneous injection" OR "subcutaneous injections" OR "cosmetic technique" OR "cosmetic techniques" OR "cosmetic technic" OR "cosmetic technics")	
ProQuest Dissertations & Theses Citation Index	TS=("adverse effect" OR "adverse effects" OR "adverse event" OR "adverse events" OR "adverse reaction" OR "adverse reactions" OR complication OR complications OR nodule OR nodules OR papule OR papules OR dyschromia OR "vascular compression" OR edema OR dropsy OR hydrops OR anasarca OR contusion OR contusions OR bruise OR bruises OR hematoma OR hematomas OR erythema OR erythemas OR ecchymosis OR ecchymoses OR pruritus OR pruritus OR itching OR itchings OR pain OR pains OR ache OR aches OR "physical suffering" OR ulcer OR ulcers OR infection OR infections OR exanthema OR exanthemas OR rash OR rashes OR keloid OR keloids OR granuloma OR granulomas OR cellulitis OR cellulitides OR necrosis OR necroses OR blindness OR "vision loss" OR anaphylaxis OR anaphylaxes OR hypersensitivity OR hypersensitivities OR allergy OR allergies OR "allergic reaction" OR "allergic reactions") AND TS=("polydioxanone" OR "PDO filler" OR "Ultra V" OR "ULTRACOL" OR "poly-D,L-lactic acid" OR "PDLLA" OR "AestheFill" OR "Durapatite" OR "Hydroxyapatite" OR "Hydroxylapatite" OR "Interpore 200" OR "Interpore200" OR "Interpore 500" OR "Interpore500" OR "Alveograf" OR "Calcitite" OR "Osprovit" OR "Ossopan" OR "Osteogen" OR "Periograf" OR "polycaprolactone" OR "poly lactide" OR "poly-L-lactic acid" OR "PLLA" OR "polylactic acid" OR "Radiesse" OR "rennova" OR "ellanse" OR "harmonyca" OR "Sculptra" OR "Elleva" OR "Diamond" OR "collagen biostimulator" OR "collagen biostimulators" OR collagen) AND TS=("dermal filler" OR "dermal fillers" OR "skin filler" OR "skin fillers" OR "subcutaneous injection" OR "subcutaneous injections" OR "cosmetic technique" OR "cosmetic techniques" OR "cosmetic technic" OR "cosmetic technics")	17
Google Scholar	("adverse effects" OR "adverse events" OR "adverse reaction" OR complication OR complications OR nodule OR nodules OR papule OR dyschromia OR "vascular compression" OR edema OR dropsy OR hydrops OR anasarca OR contusion OR bruise OR hematoma OR erythema OR ecchymosis OR pruritus OR itching OR pruritis OR pain OR ache OR aches	200

	OR "physical suffering" OR ulcer OR infection OR exanthema OR rash OR keloid OR granuloma OR cellulitis OR necrosis OR blindness OR "vision loss" OR anaphylaxis OR hypersensitivity OR hypersensitivities OR allergy OR allergies OR "allergic reaction" OR "allergic reactions") AND ("Hydroxyapatite" OR "polycaprolactone" OR "poly lactide" OR "poly-L-lactic acid" OR "PLLA" OR "polylactic acid" OR "Radiesse" OR "collagen biostimulator" OR "polydioxanone" OR "PDO filler" OR "poly-D,L-lactic acid") AND ("dermal filler" OR "skin filler" OR "subcutaneous injection" OR "cosmetic technique" OR "cosmetic technic")	
